# Redefinition of Synovial Fibroblasts in Rheumatoid Arthritis

**DOI:** 10.14336/AD.2024.0514

**Published:** 2025-07-16

**Authors:** Yinci Zhang, Xiong He, Dongdong Yin, Yihao Zhang

**Affiliations:** ^1^First Affiliated Hospital of Medical School, Anhui University of Science and Technology, Huainan, China.; ^2^Department of health inspection and quarantine, School of Public Health, Anhui Medical University, Hefei, China.; ^3^School of Pharmacy, Anhui Medical University, Hefei, China.

**Keywords:** Rheumatoid arthritis, Heterogeneity, Immune cells, Innate immunity, Adaptive immunity, Synovial fibroblast subsets

## Abstract

The breakdown of immune tolerance and the rise in autoimmunity contribute to the onset of rheumatoid arthritis (RA), driven by significant changes in immune components. Recent advances in single-cell and spatial transcriptome profiling have revealed shifts in cell distribution and composition, expanding our understanding beyond molecular-level changes in inflammatory cytokines, autoantibodies, and autoantigens in RA. Surprisingly, synovial fibroblasts (SFs) play an active immunopathogenic role rather than remaining passive bystanders in RA, with notable alterations in their subpopulation distribution and composition. This study examines these changes in SF heterogeneity, assesses their impact on RA progression, and elucidates the immune characteristics and functions of SF subsets in the RA autoimmunity, encompassing both intrinsic and adaptive immunity. Additionally, this review discusses therapeutic strategies targeting immune SF subsets, highlighting the potential of future interventions in SF phenotypic reprogramming. Overall, this review redefines the role of SFs in RA and suggests targeting SF phenotypic reprogramming and its upstream molecules as a promising therapeutic approach to restore immune balance and modulate immune tolerance in RA.

## 1. Introduction

Rheumatoid arthritis (RA) is a chronic systemic autoimmune condition marked by irreversible joint damage and heightened systemic immune activity [1-3]. Loss of immune tolerance and the development of aggressive autoimmune reactions are pivotal in RA pathogenesis, driven by substantial alterations in immune components [4-6]. While diagnostic and monitoring technologies have advanced understanding of RA, the precise pathogenic changes in immune components and mechanisms of autoimmune response remain unclear, underscoring the need to reassess our comprehension of RA and pursue new discoveries.

In recent decades, the focus on immune component alterations in RA has predominantly centered on inflammatory cytokines, autoantibodies, and autoantigens, etc [1-3]. However, there has been a notable lack of attention toward changes in cellular components, particularly within the joint synovium. Profound alterations in the cellular composition of the joint synovium, marked by the development of ectopic germinal centers and pannus, aberrant proliferation of synoviocytes, and extensive infiltration of inflammatory cells, are key drivers of RA progression [1-6]. This highlights the critical need for a more thorough understanding of the chronological and spatial pathogenic progression of synovial cellular components in RA.

Synovial fibroblasts (SFs) are key cellular components driving the transition of the joint synovium from a healthy to a pathological state [7]. Traditionally viewed as passive in the RA autoimmunity due to their lack of immune phenotypes, SFs have recently been recognized for their heterogeneity and the spatiotemporal patterns of their pathological evolution revealed by single-cell and spatial transcriptome profiling [8-19]. Various subsets of SFs exhibit robust immune properties that influence cellular, humoral, and innate immunity, thereby contributing significantly to RA progression [9-19]. Additionally, these SF subsets display a secretory phenotype for cytokines, which in turn can activate both adaptive and innate immune cells [20-24]. Therefore, reassessing the characteristics of SFs and their actual role in RA is crucial.

This review systematically summarizes the immunophenotypes and properties of SF subsets, exploring their contributions to the RA autoimmunity from both innate and adaptive immunity perspectives. Additionally, it comprehensively evaluates the therapeutic impacts of targeting immune SF subsets and underscores the potential of future strategies involving SF phenotypic reprogramming. In summary, this review redefines the roles of SFs in RA autoimmunogenesis and the specific pathology of joint involvement.

## 2. Transformation of SFs from physiological to pathogenic states in early RA

Compared to other synovial cells, SFs exhibit heightened phenotypic and functional plasticity during RA progression. The shift of SFs from a physiological to a pathological phenotype is a crucial event in the early stages of RA. This transformation involves a temporal and spatial evolutionary process, ultimately resulting in irreversible synovial changes like hyperplasia, ectopic germinal center development, and pannus formation.

The synovium is a thin mesenchymal membrane composed of a synovial lining layer near the joint cavity and a synovial sublining layer, both populated by SFs with distinct characteristics. SFs in the lining layer are equipped with abundant rough endoplasmic reticula and ribosomes, which are crucial for secreting hyaluronic acid and proteoglycans to regulate synovial fluid balance. In contrast, SFs in the sublining layer exhibit higher proliferation rates but do not actively secrete synovial fluid solutes. These mesenchymal cells vary in origin and biological functions based on their synovial location, crucial for maintaining synovial function and joint stability under normal conditions. During early RA, SFs undergo a pathological transformation, initiating inflammatory processes that lead to joint damage. Activated SFs demonstrate two distinct pathological traits: potent immune-mediated capabilities and heightened aggressiveness [8-19]. These characteristics categorize activated SFs into two distinct clusters, each occupying specific regions within the synovial membrane of joints. This heterogeneity among SFs underscores their diverse biological roles and origins.

**Figure 1.**
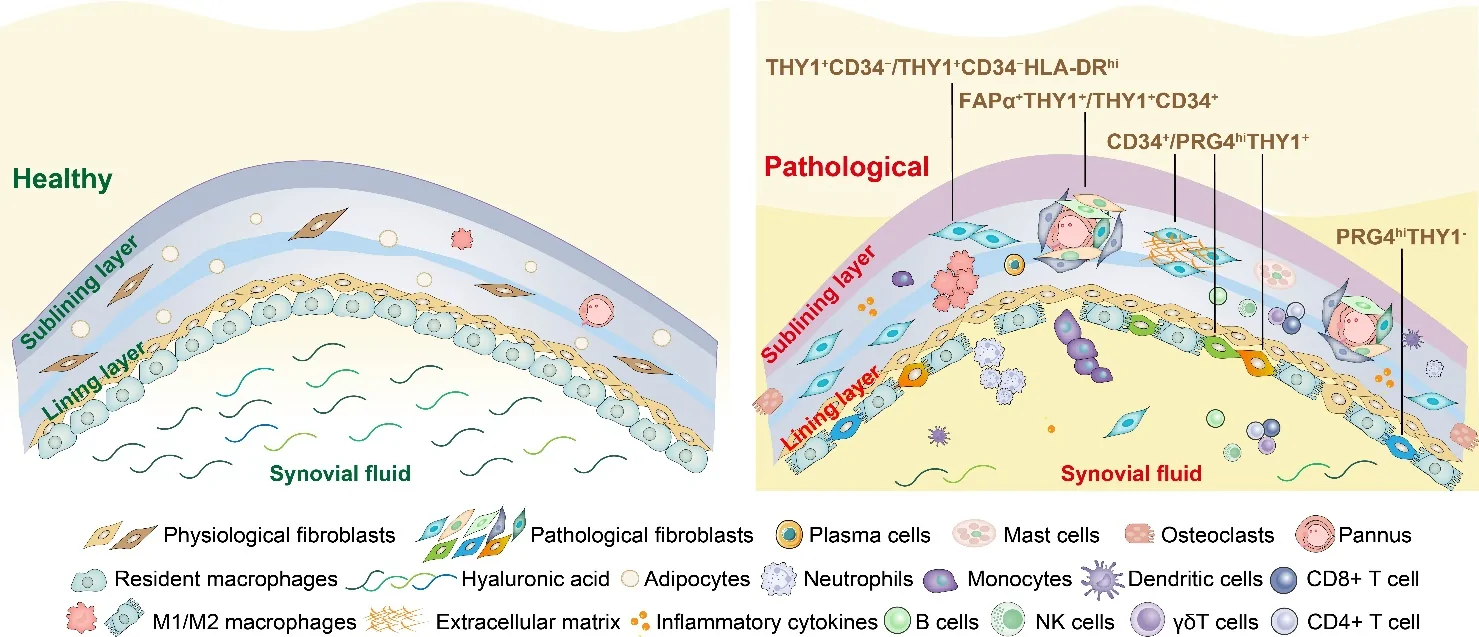
**Transformation of SFs from physiological to pathological states in RA**. The synovium in its healthy state comprises a thin interstitial membrane containing two types of SFs: those in the synovial lining layer near the joint cavity and those in the subsynovial layer. These SFs remain dormant and are vital for maintaining synovial function and joint stability. However, in RA, SFs undergo a pathological activation, shifting from their normal roles to become pathogenic. This transformation involves a notable expansion and repositioning of SF subpopulations within the synovial membrane. Expanded SF subpopulations secrete increased levels of inflammatory factors and chemokines, leading to the accumulation of inflammatory immune cells and initiating RA-related inflammation. During RA flares, abnormal SF subpopulation expansion results in synovial thickening, heightened matrix production, and reduced synovial fluid, ultimately contributing to joint damage.

Subsets of fibroblasts within the synovial sublining expand significantly and play a critical role in RA [8-11]. However, the initial phenotypic changes in RA-SFs occur before substantial inflammation begins, driven by innate immune mechanisms. Essentially, SFs proliferate in response to innate immune signals before inflammatory cells migrate to the synovium, adhere to joint surfaces, and invade, initiating cartilage destruction [25-30]. In contrast, abnormal SF proliferation leads to the excessive production of inflammatory factors and chemokines, contributing to the inflammation seen in RA [20-22,32-34]. Certain SF subsets with expanded distribution perpetuate the chronic inflammation of RA by contributing to the activation of innate, cellular, and humoral immunity [9-19]. During inflammatory phases, the abnormal expansion of SFs causes thickening of the synovium and increased vascularization [35]. Endogenous activation of SFs also promotes the accumulation of inflammatory cells like macrophages and lymphocytes, while inhibiting their apoptosis, which is crucial for the persistent synovial tissue hyperplasia in RA [36-39].

In summary, as depicted in Figure 1, the transformation of SF characteristics from a physiological to a pathological state represents their transition from mesenchymal cells to immune cells, accompanied by a redistribution of SF subset proportions in RA. Hence, it is crucial to thoroughly examine the redistribution of diverse SF populations in RA and its association with disease progression.

## 3. Redistribution of the proportions of heterogeneous SFs in RA and their correlation with disease status

Recent advancements in single-cell mapping, spatial transcriptome analysis, and epigenome sequencing have identified distinct subsets of fibroblasts [8-19]. These technologies have revealed dynamic changes in fibroblast subsets across various inflammatory conditions [8-19], challenging the traditional view of fibroblasts as uniform and highlighting the diverse roles played by different subsets in disease progression [8-19]. This review summarizes the shifting proportions of heterogeneous fibroblast subsets in RA and their correlation with disease outcomes (Table 1). Additionally, it is noteworthy that different fibroblast subsets reside in specific anatomical sites [8-11], suggesting that changes in fibroblast phenotypes are accompanied by alterations in subset proportions at these sites. Therefore, analyzing fibroblast subsets within their anatomical contexts is crucial for understanding the site-specific mechanisms involved in RA pathogenesis.

**Table 1:** Redistribution of cell proportions of RA-SF subsets and its correlation with clinical disease status.

RA-SFs subsets	Species	Localization	Proportion	Pathological action	References
Dkk3/Lrrc15^+^; Birc5/Aqp1^+^ (PRG4^hi^THY1^+^)	Mice (Overexpression of human TNF induced RA model)	Both lining and sublining	More abundant in RA at 25% and 14% compared to 2% and 0.17% in healthy Joints	Enhanced inflammatory responses and matrix catabolic processes	Armaka et al, 2022
PRG4^hi^/Tspan15^+^ (PRG4^hi^THY1^-^)		Lining	Increase in the proportion of cell numbers in RA compared to healthy joints	Cell expansion is accompanied by some loss of homeostatic function during RA progression	
Smoc2/Col15a1^+^; Pxt3/Notch3^+^; Comp/Sfrp1^+^; Osr1/Nr2f2^+^; Meox1/Clu^+^; Dpp4/Pi16^+^ (PRG4^low/-^THY1^+^)		Sublining	Decreased percentage of cells in RA compared to healthy joints	Promotes tissue repair, osteogenesis and cartilage formation, maintains normal joint morphogenesis, participates in vascular remodeling, and immune surveillance	
PRG4^+^	Human (n = 9 RA & 11 OA patients)	Lining	More abundant in OA at 48% compared to RA	N/D	Wei et al, 2020
THY1^+^		Sublining	More abundant in RA at 52% compared to 31% in OA	Causes inflammation and swelling in RA joints	
FAPα^+^THY1^+^	Mouse (Serum transfer induced RA model)	Sublining	More abundant in RA compared to OA and the proportion of cells increased with increasing severity of joint inflammation and bone erosion	Causes more severe and persistent inflammatory arthritis with minimal impact on bone and cartilage	Croft et al, 2019
FAPα^+^ THY1^-^		Lining	More abundant in OA compared to RA and the proportion of cells increased with increasing severity of cartilage damage	Mediates bone and cartilage damage with little effect on inflammation	
THY1^+^CD34^+^	Human (n = 36 RA & 15 OA patients)	Sublining	More abundant in RA compared to OA	Causes more severe and persistent inflammatory arthritis	Zhang et al, 2019
THY1^+^CD34^-^HLA-DRA^hi^		Sublining	Expanded >15-fold in RA compared to synovial tissue in OA (36% versus 2%); It has the highest abundance in the subset	Causes more severe and persistent inflammatory arthritis	
THY1^+^DKK3^+^		Sublining	More abundant in OA compared to RA	Prevents cartilage degradation *in vitro*	
THY1^-^CD55^+^		Lining	More abundant in OA compared to RA	High expression of genes detrimental to RA progression	
CD34^-^THY1^-^	Human (n = 16 RA & 26 OA patients)	Lining	Less abundant in RA at 15% compared to 48% in OA	Less abundant in the swollen joints compared to non-swollen joints; Promotes osteoblast differentiation	Mizoguchi et al, 2018
CD34^-^THY1^+^		Sublining	More abundant in RA at 22% compared to 8% in OA	Increased inflammation and joint swelling in RA joints; Promotes osteoblast differentiation	
CD34^+^		Both superficial lining and deeper sublining	More abundant in the swollen joints compared to non-swollen joints	In the recruitment of monocytes in inflamed synovial tissue	

Note: RA, rheumatoid arthritis; SFs, synovial fibroblasts; TNF, tumor necrosis factor; OA, osteoarthritis; N/D, no data.

Mizoguchi et al. [11] utilized single-cell RNA sequencing to classify three SF subsets in a study involving 26 osteoarthritis (OA) patients and 16 RA patients: cluster of differentiation 34 (D34)^-^Thy-1 cell surface antigen Gene (THY1, or CD90)^-^ for the lining layer, CD34^-^THY1^+^ for the deep sublining layer, and CD34^+^ for both layers. It was discovered that the frequency of CD34^-^THY1^-^ fibroblasts was lower in RA patients (15%) compared to OA patients (48%), and this subset was less prevalent in swollen joints than in nonswollen joints. Conversely, CD34^-^THY1^+^ fibroblasts were found more frequently in RA (22%) than in OA (8%), and more so in swollen joints. Similarly, CD34^+^ fibroblasts were predominantly found in swollen joints. Zhang et al. [10] identified four potential SF subsets by combining single-cell transcriptomics with mass cytometry: CD34^+^, major histocompatibility complex, class II, DR alpha (HLA-DRA)^hi^, dickkopf3 (DKK3)^+^, and CD55^+^, with the first three being THY1^+^ clusters indicative of sublining fibroblasts, while the fourth represented lining fibroblasts. An increase in the populations of CD34^+^ and HLA-DRA^hi^ fibroblasts, particularly noted in RA patients, correlated with more severe and persistent inflammation, alongside a reduction in DKK3^+^ and CD55^+^ fibroblasts. In 2019, Croft et al. [9] determined through flow cytometry that the fibroblast activation protein-α (FAPα)^+^THY1^+^ subset (sublining layer) was more prevalent in RA than in OA, and its abundance increased with greater severity of joint inflammation and bone erosion. In contrast, the FAPα^+^THY1^-^ subset (lining layer) was more prevalent in OA and increased with worsening cartilage damage. Using single-cell RNA sequencing, Wei et al. [8] divided SFs into two subsets and synovial tissue organoids: proteoglycans (PRG4)^+^ (lining) and THY1^+^ (sublining). Flow cytometry analysis showed a dominance of fibroblasts in the lining in 48% of OA patients, while RA synovium saw an expansion of sublining fibroblasts from 31% to 52%, which exacerbated inflammation and joint swelling in RA. Recently, Armaka et al. [12] identified seven steady-state SF subsets and two disease-specific subgroups using single-cell transcriptomes and epigenomes—Smoc2/Col15a1^+^ (S1), Comp/Sfrp1^+^ (S2a), Osr1/Nr2f2^+^ (S2b), Meox1/Clu^+^ (S2c), Dpp4/Pi16^+^ (S3), PRG4-high/Tspan15^+^ (S4a), and Pxt3/Notch3^+^ (S5), along with Dkk3/leucine-rich repeat-containing protein 15 (Lrrc15)^+^ (S2d) and Birc5/Aqp1^+^ (S4b). In RA mice joints, the proportions of S2d and S4b cells gradually increased from barely detectable levels (2% and 0.17%) to 25% and 14%, respectively, leading to enhanced joint inflammation and destruction. As the disease progresses, the expansion of S4a cells is associated with the loss of homeostatic functions, including the maintenance of endoplasmic reticulum calcium homeostasis and oxygen level responses, with the homeostatic subgroup hS4a in S4a being gradually replaced by the inflammatory subgroup iS4a. Moreover, Friscic et al. [15] reported that heterogeneous reprogramming of SFs, as evidenced through transcriptome and epigenome analysis, as well as targeted genetic and pharmacological studies, mediates the onset of synovial tissue inflammation.

The evidence presented indicates that alterations in the distribution of RA-SF subsets correlate with both molecular and clinical levels of tissue inflammation (Table 1). The sustained inflammation plays a pivotal role in the development of RA, underscoring the need for deeper exploration into the association between SF subsets and synovial inflammatory conditions.

## 4. SF subsets with abnormally high cell ratios maintain persistent inflammation

As previously noted, the roles of SFs vary depending on their anatomical location, subpopulation, and the clinical state of RA. Moreover, an increased representation of SF subsets within the synovial sublining layer is linked to a higher prevalence of RA [8-11]. This section will detail five specific SF subsets that are prominently involved in sustaining joint inflammation, namely FAPα^+^THY1^+^, THY1^+^CD34^-^/THY1^+^CD34^-^HLA-DR^hi^, CD34^+^/THY1^+^ CD34^+^, PRG4^hi^THY1^+^, and PRG4^hi^THY1^-^ (Table 2).

**Table 2:** Immune- and inflammation-mediated roles of SFs in RA (the role of an immune effector capable of maintaining inflammation) and their corresponding major cell subsets.

Subsets	Technical approaches	Species	Immune properties	Localization	Markers	References
**FAPα^+^THY1^+^**	Bulk and scRNA-seq	Mouse	High expression of chemokines and cytokines including: IL-6, Lif, IL-33 and IL-34; Increased effector CD4^+^ T cells, decreased Foxp3^+^ Tregs, and a global increase in neutrophil and macrophage infiltration	Sublining layer	FAPα, THY1, RANKL, MMPs, CCL9,	Croft et al, 2019; Floudas et al, 2022
**THY1^+^CD34^-^/THY1^+^CD34^-^HLA-DR^hi^**	Bulk and scRNA-seq, mass cytometry	Human	High expression of chemokines and cytokines including: IL-6, CXCL12 and HLA-DRA; Express genes related to MHC class II presentation and the IFNγ-mediated signaling pathway; Mediating the recruitment of immune cells	Sublining layer (perivascular)	CXCL12, CCL2, ADAMTS1, THY1, HLA-DRA, MMP-13, Collagen genes, POSTN	Zhang et al, 2019; Mizoguchi et al, 2018; Micheroli et al, 2022; Alivernini 2020
**CD34^+^/THY1^+^CD34^+^**	Bulk and scRNA-seq	Human/ Mouse	High expression of chemokines and cytokines including: IL-6, CXCL12 and CCL2; Recruiting more peripheral blood mononuclear cells; Immunoregulatory, tissue priming via C3 activation, proinflammatory effect	Superficial lining and deeper sublining areas of thesynovium	C3, CD34, THY1, Apod, Mfap5, Clip	Mizoguchi et al, 2018; Friščić et al, 2021; Micheroli et al, 2022; Alivernini 2020; Julià et al, 2020
**PRG4^hi^THY1^+^**	scRNA-seq and scATAC-seq	Mouse	Expression of Mki67, Pdgfa, Birc5, Aqp1, Acta2, C1qtnf3 adipokines and other chemokines, such as CXCL5, as well as several adhesion molecules, actively contribute to the arthritic inflammatory process	Both lining and subling layer	Dkk3, Lrrc15, Birc5, Aqp1, Fbln7, Thbs4, Cthrc1, Runx1, Mki67,Pdgfa,Birc5,Aqp1,Acta2,C1qtnf3, CXCL5	Armaka et al, 2022
**PRG4^hi^THY1^-^**	scRNA-seq and scATAC-seq	Mouse	Expression of markers of inflammatory response (CCL2, CCL5, Hmox1, Saa3), class I antigen presentation (H2-K1, B2m, H2-Q7) and ECM remodeling (MMP-3, Timp1, CD44)	Lining layer	Tspan15, Hbegf, Htra4, CD55, MMP-3, FN1	Armaka et al, 2022; Micheroli et al, 2022

Note: SFs, synovial fibroblasts; RA, rheumatoid arthritis; ECM, extracellular matrix.

### 4.1 FAPα^+^THY1^+^ SF subset

Croft et al. [9], using flow cytometry, observed that the FAPα^+^ subset can enhance the progression of various arthritis states in mice by intensifying inflammation and exacerbating bone erosion. Consistent with predictions from single-cell transcriptome analysis, the FAPα^+^THY1^+^ subset demonstrated heightened expression levels of cytokines and chemokines such as interleukin (IL)-6, IL-33, and IL-34 [9]. In mouse models, injection of podoplanin (PDPN)^+^FAPα^+^THY1^+^ cells induced infiltration of CD4^+^ T cells and macrophages, contributing to immune effects, while promoting regulatory T cells (Tregs) expressing Foxp3 and showing a relative reduction in neutrophil counts [9]. Furthermore, a greater proportion of the PDPN^+^FAPα^+^THY1^+^ subset was found in the synovium of RA patients with persistent joint inflammation compared to OA patients, accompanied by increased secretion of inflammatory factors and expression of systemic inflammatory markers [9]. Overall, the PDPN^+^FAPα^+^THY1^+^ subset assumes an immunoinflammatory regulatory role through production of diverse chemokines and cytokines that sustain chronic inflammatory responses.

Floudas et al. [13] observed distinct distributions of SF subsets through single-cell transcriptomic profiling, noting a significant enrichment of the FAPα^+^THY1^+^ subset in the sublining layer of RA synovium, while being nearly absent in the lining layer. In synovial tissue, macrophages constitute the predominant immune cell population, playing dual roles in protection and inflammation during RA pathogenesis [40-42]. Among the three identified macrophage clusters in RA synovial tissue, subset 1, characterized by high expression of IL-1β, likely plays a pivotal role in promoting inflammation [13]. Notably, the FAPα^+^THY1^+^ subset expressed elevated levels of the IL-1β receptor IL-1R1 [13]. Furthermore, specific T-cell populations in RA patients exhibit heightened expression of immunomodulatory transforming growth factor beta 1 (TGF-β1). The transcriptional profile of the proinflammatory FAPα^+^THY1^+^ subset is influenced by TGF-β1 derived from synovial T-cells and IL-1β from macrophages [13]. Synergistically, IL-1β and TGF-β1 contribute to metabolic adaptations, promote secretion of the proinflammatory marker IL-6, and upregulate intercellular cell adhesion molecule-1 (ICAM-1) expression, thereby facilitating tissue invasion and enhancing immune cell adhesion within RA-SFs [13].

In the studies cited, FAPα^+^THY1^+^ cells were found to express molecular markers, including receptor activators for receptor Activator for Nuclear Factor-κ B Ligand (RANKL), matrix metalloproteinases (MMPs), chemokine (C-C motif) ligand (CCL)9 [9,13]. Together, these findings indicate that the proportion of the FAPα^+^THY1^+^ subset in the sublining layer is notably increased in RA individuals, contributing to sustained inflammation that drives the progression of RA.

### 4.2 THY1^+^CD34^-^/THY1^+^CD34-HLA-DR^hi^ SF subset

Mizoguchi et al. [11], using single-cell RNA sequencing, identified a CD34^-^THY1^+^ SF subset that exhibits high proliferative and invasive capabilities and secretes proinflammatory cytokines. They demonstrated an expansion of this subset in RA patients, distinguishing it from OA. Notably, CD34^-^THY1^+^ RA-SFs were observed to proliferate near lymphocyte aggregates [11]. Additionally, RANKL expression was found to be elevated in this subset, contributing to T-cell trafficking during autoimmune inflammation [43]. Therefore, the CD34^-^THY1^+^ subset likely plays a pathogenic role in RA by recruiting immune cells. Conversely, an increased proportion of CD34^-^THY1^+^ fibroblasts promote leukocyte infiltration, exacerbating synovitis and synovial hypertrophy [11]. Thus, the progression of active RA involves immune cell infiltration driven by the expansion of CD34^-^THY1^+^ fibroblasts. Integrating published single-cell RNA sequencing data, Micheroli et al. [14] demonstrated high expression of chemokine (C-X-C motif) ligand (CXCL)12 and HLA-DRA in the CXCL12^+^ SF subset, while CD34 expression was absent. Consistent with previous studies [9,10], genes associated with inflammatory responses in this subset were more highly expressed in RA joints rich in leukocytes compared to OA joints. This suggests a significant presence of HLA-DRA^hi^CD34^-^THY1^+^ fibroblasts in RA synovium. Notably, CD34 serves as a marker for hematopoietic stem cells [44,45] and is used to isolate mesenchymal stem cells by negative selection. Together, these findings indicate that CD34^-^THY1^+^ fibroblasts play dual roles as recruiters and targets in RA, acting akin to proinflammatory immune cells within the joint environment.

Additionally, Zhang et al. [10] identified a CD34^-^THY1^+^HLA-DR^hi^ SF subset in the synovial sublining layer that exhibited significantly greater proliferation in leukocyte-rich RA compared to OA, using integrated single-cell transcriptomics and mass cytometry. This subset expresses genes associated with major histocompatibility complex (MHC) class II and interferon (IFN)γ-mediated signaling pathways [10], indicating upregulation of MHC class II molecules in response to IFNγ signaling. Increased abundance of the CD34^-^THY1^+^HLA-DR^hi^ SF subset correlated positively with elevated expression of IL-6, CXCL12, and HLA-DRA [10]. Pathway analysis of RA samples revealed co-expression of interferon-responsive genes in SFs alongside monocytes and T cells [10], suggesting intercellular communication within the RA synovium. However, there was no significant differential expression of genes related to inflammatory responses between RA samples infiltrated by leukocytes and those without such infiltration [10]. Furthermore, IL-6 expression was predominantly observed in CD34^-^THY1^+^HLA-DR^hi^ SFs and B-cell subsets [10]. Hence, it is hypothesized that in the synovium of RA individuals, SFs contribute to inflammation expansion not only through interactions with traditional immune cells but also via autoimmune mechanisms, thereby contributing to the progression of RA.

### 4.3 CD34^+^/CD34^+^THY1^+^ SF subset

Mizoguchi et al. [11] identified CD34^+^ fibroblasts through single-cell RNA sequencing, demonstrating their enrichment and significantly enhanced proliferation, migration, and invasive capabilities in swollen joints. SFs primarily function as chemokine producers and recruiters of leukocytes to inflamed tissues [46,47]. CD34^+^ fibroblasts are characterized by high secretion of IL-6, CXCL12, and CCL2, which facilitate monocyte recruitment to inflamed synovial tissue, thereby amplifying inflammation [11]. Inflammatory sensitization of fibroblasts occurs gradually through repeated or sustained inflammatory stimuli [48]. Friscic et al. [15], through transcriptome and epigenome analysis, revealed that the CD34^+^THY1^+^ subset expresses multiple complement components that contribute to tissue inflammation, known to exacerbate RA deterioration [49,50]. Interestingly, T cells can also produce complement components C3 and C5 and their receptors, activating them [51]. Targeting C3 or C3a in RA has shown more promising outcomes compared to directly targeting effector cytokines or exogenous complement factors [15]. These findings suggest that the CD34^+^THY1^+^ SF subset may exhibit immune functions akin to T cells in RA, sustaining inflammation and promoting disease progression. Conversely, the CXCL14^+^ SF subset, characterized by high levels of CXCL14, C3, asporin (ASPN), THY1, and CD34 [14], showed a negative association with RA activity. ASPN, known to inhibit TGF-β function and promote OA pathogenesis, may contribute to this effect [14]. However, Mizoguchi et al. [11] cultured SF subsets, potentially altering their behavior. Alivernini et al. [19], utilizing single-cell transcriptomics, separated CD34^+^ and CXCL14^+^ subsets and found that CXCL14^+^ cells express high levels of growth arrest-specific protein 6, which regulates macrophage function in resolving synovial tissue, suggesting an anti-inflammatory role for this SF subset. This study underscores that the biological properties and functions of SFs are determined by their specific phenotypes.

Aberrant activation of phosphatidylinositol-4,5-bisphosphate 3-kinase, catalytic subunit alpha (PIK3CA), PIK3CB, and the leukocyte signaling kinase PIK3CD in pathological signaling pathways is implicated in the progression of various diseases [54,55]. Julià et al. [16], analyzing the synovial transcriptome, demonstrated that B cells differentiate into different isoforms upon activation, producing a series of PIK3CD genes whose downregulation correlates with improved treatment response. While PIK3CA and PIK3CB are broadly expressed in human tissues, PIK3CD is specifically expressed in immune system cells [56-58]. Recent studies have highlighted high expression of PIK3CD in RA-SFs [59]. Moreover, tumor necrosis factor (TNF) induces elevated PIK3CD expression in SFs but not in leukocytes [60]. Interestingly, Julià et al. [16] reported a significant increase in CD34^+^ fibroblasts in response to anti-TNF therapy. These findings suggest that expanded SF subsets in RA exhibit immune-like functions that contribute to the progression of the disease.

### 4.4 PRG4^hi^THY1^+^ SF subset

Armaka et al. [12] found that THY1 and PRG4 were primarily expressed by distinct SF subsets in healthy joint tissue, but in RA, especially in disease-enriched S2d (Dkk3/Lrrc15^+^) and S4b (Birc5/Aqp1^+^) SFs, they were more likely to be coexpressed across both the sublining and lining compartments. The amplified S2d SF subset in RA expresses genes critical for joint pathology, including the extracellular matrix component fibulin 7 (Fbln7), vascular remodeler collagen triple helix repeat containing 1 (Cthrc1), Wnt inhibitor Dkk3, novel mesenchymal protein Lrrc15, and transcription factor Runx1 [12]. Fbln7 is a secreted glycoprotein with antiangiogenic and immunomodulatory effects, influencing functions of monocytes, macrophages, and neutrophils, thereby impacting inflammation [61,62]. Cthrc1 exhibits immunosuppressive effects and promotes infiltration of various immune cells [63]. Dkk3 functions as an immune tolerance regulator in peripheral CD8^+^ T cells [64], while Lrrc15 myofibroblasts establish the stromal setpoint for inhibiting tumor immunity [65]. Additionally, Hsu et al. [66] demonstrated that Runx1 is crucial for T-cell maturation, with its deletion leading to initial blockage of intrathymic CD4 T-cell maturation, suggesting involvement in immune regulation. Conversely, the S4b SF subset actively contributes to arthritis inflammation by expressing the proliferation marker ki-67 (Mki67), platelet-derived growth factor subunit a (Pdgfa), baculoviral IAP repeat containing 5 (Birc5), aquaporin 1 (Aqp1), actin alpha 2 (Acta2), C1q, and TNF-related 3 (C1qtnf3) adipokines, alongside high THY1 and PRG4 expression [12]. These findings underscore that the PRG4^hi^THY1^+^ SF subset may enhance RA responses through immune-mediated effects.

### 4.5 PRG4^hi^THY1^-^ SF subset

Armaka et al. [12] demonstrated that S4a (PRG4^hi^/ Tspan15^+^) SFs in the lining layer, lacking THY1 expression, exhibit high levels of PRG4 along with other genes such as tetraspanin 15 (Tspan15) and HtrA serine peptidase 4 [9,10]. These S4a SFs maintain some markers of homeostasis during TNF-α-mediated arthritis but show increased transcriptome diversity, indicating potential compromise in their reparative function post-disease onset [12]. Additionally, markers of inflammation (CCL2, CCL5, heme oxygenase 1, serum amyloid a 3), class I antigen presentation (histocompatibility 2 (H2)-K1, beta-2-microglobulin, H2-Q7), and extracellular matrix remodeling (MMP3, TIMP metallopeptidase inhibitor 1 (Timp1), CD44) were detected in these SFs [12], consistent with previous studies on lining SFs in arthritic individuals [9,10]. Analysis of SF subset enrichment across different pathologies [14] revealed an amplified ratio of PRG4^+^THY1^-^ SFs in myelopathological and lymphoid types compared to rhabdomyosarcoma types, indicating strong influence from immune cells on PRG4^+^ gene expression in synovial membranes involved in arthritis development [14]. These findings expand on the functional characterization of PRG4^+^ SF subsets proposed by Wei et al. [8], emphasizing their immune effector properties in RA.

## 5. The role of SFs in the immune system

SFs undergo a significant functional shift as RA progresses, transitioning from acting as immune-suppressors to becoming active immunostimulators [67-70]. The increase in SF subsets with immune-mediated effects suggests they have a positive role in stimulating the immune response and actively driving RA pathogenesis, leading to the evolution of synovitis from acute inflammation to chronic tissue destruction (Table 3). Chronic synovitis exerts pressure on both traditional immune cells, potentially amplifying disease severity [71]. However, the precise immune role of SFs, including whether they fulfill traditional immune cell functions, remains unclear. This section systematically explores the involvement of SFs in the immune system of RA (Table 3).

**Table 3:** The role of SFs in the immune system in RA.

Immune function	Finding	Effector	References
**Involved in inherent immunee**	Highly expressed PRRs and complement receptors	TLR2, TLR3, TLR4, TLR7, TLR9, NOD-1, NOD-2, C3aR and C5aR	72-80
	Release immune cytokines, including inflammatory cytokines and complements	IL-6, IL-8, CCL5, IFNγ, IL-17, TNF-α, IL-1β, CXCL9, CXCL10, CXCL11, C1r, C1s, C2, complement factor B, complement factor H, C3, C3a and C5b-9	20, 81-87
	Induces inherent immune cell infiltration	macrophages, DCs, NK cells, NKT cells, granulocytes and mast cells	14, 88-90, 91-93
**Involved in cellular immunity**	Release immune cytokines	IL-2, IL-4, IL-6, IL-8, IL-7, IL-15, IL-17, IL-33 and IL-34	14-16, 19-21, 24, 94-96, 97-100
	Induces T lymphocyte infiltration and activation	CXCL10, IFNγ, TNF-α, Th1 and CD4^+^ T cells	14, 101-103
	Delivery of antigen to CD4^+^ T cells via MHC-II as an antigen-presenting cell to assist CD4^+^ T cells in mediating the immune response	MHC-II molecules	85, 104-108
	Strong response to IFNγ pro-inflammatory molecules secreted in large quantities by CD8^+^ T cells; Enhanced crosstalk with Th cells; Mutual activation with CCR6^+^ memory Th cells in a pro-inflammatory feedforward loop	IFNγ, MMP-3, IL-6, IL-10, CCR6^+^, CD8^+^ T cells and Th cells	15, 101, 109-111
**Involved in humoral immunity**	High expression of B-cell surface antigens and receptors	CD16, CD40, CD80, CD90, CD95, ICAM-1, MHC-II molecules, SLAMF8, IL-1R, IL-6R and IgDR	15, 16, 117-121
	Induce B-cell infiltration, assist B-cell activation, and promote B-cell and plasma cell maturation, expansion and survival	BAFF, IL-6, IL-15, Mig/CXCL9, hVCAM1 (CD106), osteopontin, fibronectin, B cells and plasma cells	18-24, 124-131
	Interaction with activated B cells leads to conversion of non-arthritic SFs into SFs with a proinflammatory and aggressive RA-like phenotype; High expression of cytokines that mediate crosstalk between plasma cells and SFs	IL-36R, HIF-1α, IFNγ, IL-17, IgG, TNF-α, hVCAM1, activated B cells, plasma cells, Th1 and Th17 cells, regulatory B10 and congenital-like B cells	131-135

Note: SFs, Synovial Fibroblasts; RA, Rheumatoid Arthritis; PRRs, Pattern Recognition Receptors.

### 5.1 Function of SFs in the intrinsic immune system

During the progression of disease, innate immune cells recognize pathogen-associated molecular patterns through pattern recognition receptors (PRRs) recognition [72-76]. Historical evidence indicates that SFs can function as innate immune cells by expressing PRRs, particularly Toll-like receptors (TLRs) such as TLR2, TLR3, TLR4, TLR7, and TLR9, and nucleotide-binding and oligomerization domains (NODs) such as NOD-1 and NOD-2 [72-76]. Synergistic activation of RA-SFs occurs with NOD-1 and TLR2 ligands or NOD-1 and TLR4 ligands, leading to the secretion of IL-6, CCL5, and MMPs, which promote RA progression [73]. Polyenylphosphatidylcholine reduces synovial inflammation by inhibiting TLR2 and its mitogen-activated protein kinase and nuclear factor kappa-B (NF-κB) pathways [77]. In early RA stages, the overexpression of TLRs (especially TLR3 and TLR4) in SFs leads to persistent inflammation and joint destruction [74]. Melanoma differentiation-associated gene 5 downregulates CXCL10 expression by inhibiting TLR3 signaling in RA-SFs, alleviating synovial inflammation in RA individuals [78]. Samarium alcohol inhibits SF proliferation and invasion via the TLR4/NF-κB pathway, thereby delaying RA progression [79]. TLR7 activation promotes RA bone destruction by inducing osteoblast differentiation from their precursors and increasing RANKL production in RA-SFs [80].

Innate immune molecules such as cytokines, the complement system, defensins, and lysozyme play crucial roles in the innate immune response. Pathogen infections stimulate immune cells and infected tissues to produce diverse cytokines, triggering inflammation. Lipo-polysaccharides increase TNF-α, IL-1β, and MMP levels in RA-SFs, promoting RA progression [81]. Additionally, RA-SFs exhibit higher IL-6 and IL-8 levels compared to normal SFs [82,83]. When SFs are cocultured with T cells, TLR activation facilitates intercellular contact and cytokine-mediated amplification of T helper (Th)1 and Th17 cells, leading to IFNγ and IL-17 production [84]. RA-SFs also secrete CXCL9, CXCL10, and CXCL11, chemokines that activate chemokine receptor 3 (CXCR3) [85]. The synovium of RA patients shows elevated expression of complement components like C1r, C1s, C2, C3, complement factor B, complement factor H, C5b-9, and complement receptors C3aR and C5aR [20,86,87]. Recent studies suggest that complement C3 and C3aR activation induces local inflammatory responses by metabolically reprogramming SFs independently of the adaptive immune system [20].

SFs can additionally promote the infiltration of innate immune cells. For instance, natural killer (NK) cells and NKT cells adhere to and migrate more prominently beneath monolayers of RA-SFs compared to OA-SFs. They interact with SFs, inducing the secretion of IL-15, chemokines, and MMP [88]. Abnormal neutrophil levels in RA-SFs were reported by Liao et al. [89] and validated in clinical samples [90]. Earlier work by Nanki et al. [90] demonstrated that the chemokine fractalkine, expressed on RA-SF, contributes to the accumulation of macrophages and dendritic cells expressing these chemokine receptors. Overall, FAPα^+^THY1^+^ SFs facilitate increased neutrophil and macrophage infiltration [9]. Additionally, several studies have indicated that mast cell infiltration can be induced by inflammatory factor signaling activation in SFs [91-93].

## 5.2 Function of SFs in the adaptive immune system

### 5.2.1 Function of SFs in the cellular immune system

T lymphocytes are key effectors in cellular immunity, characterized by an inflammatory response involving monocyte infiltration, specific cytotoxicity, or both. THY1^+^CD34^-^/THY1^+^CD34^-^HLA-DR^hi^ SFs express genes associated with major MHC class II molecules and IFNγ-mediated signaling pathways, facilitating monocyte recruitment [10,11]. CD34^+^/THY1^+^CD34^+^ SFs recruit peripheral blood monocytes for inflammatory immune regulation [11,14-16,19]. These SF subsets also produce elevated levels of IL-6, IL-33, and IL-34 [9-11,14-16,19]. Additionally, TNF-α induces SFs to secrete IL-2, IL-4, IL-7, and IL-15, exacerbating the RA response [94-96]. During immune responses, T cells produce IL-6, IL-8, IL-33, and IL-34 to regulate inflammation and other immune cells [97-100]. SFs contribute to RA progression by producing chemokines such as CXCL10 and CXCL19, which promote lymphocyte recruitment [101]. Notably, CXCL10 is highly expressed in SFs of aggressive RA cases [102], and its elevated levels stimulate Th1-directed T cells, leading to IFNγ and TNF-α production and triggering CXCL10 secretion from various cells, thereby perpetuating an amplifying feedback loop that sustains the autoimmune process [103]. Furthermore, FAPα^+^THY1^+^ fibroblasts increase effector CD4^+^ T cells while decreasing Foxp3^+^ Tregs [9], suggesting SFs' role in inducing T lymphocyte infiltration and activation.

MHC class II molecules are absent in healthy joints but become abundant during the inflammatory phase of RA [104]. SFs present antigens to CD4^+^ T cells via MHC-II molecules and aid CD4^+^ T cells in mounting immune responses during RA [105,106]. Research by Kato et al. indicates that IL-17 and IFNγ regulate SFs by upregulating CD40, CD54, and MHC-II, facilitating Th1 cells in secreting IL-6 and IL-8, thereby amplifying the inflammatory response [107]. Lipocalin enhances IL-6 production by SFs, boosting T follicular helper cell responses in RA [108]. Additionally, Ueno et al. demonstrated that SFs sustain the Th1 immune response by producing CXCR3 ligands associated with Th1 cells [85]. Thus, SFs play a pivotal role in initiating and advancing inflammation in RA. CXCL10^+^CXCL19^+^ fibroblasts directly interact with T cells [101] and may resemble the highly pathogenic HLA-DRA subset, which strongly responds to IFNγ proinflammatory molecules released by CD8^+^ T cells [10]. Hypoxia significantly impacts SFs' crosstalk with Th cells, altering RA pathophysiology by reducing SFs' ability to restrain Th cell proliferation, decreasing MMP-3, IL-6, IL-10, and IFNγ expression, and increasing IL-17A levels [109]. This study indirectly suggests that SFs and Th cells interact to trigger immune responses that promote RA progression. Moreover, the activation of both chemokine C-C-subunit receptor 6^+^ memory Th cells and SFs in a proinflammatory feedback loop has been observed, potentially perpetuating synovial inflammation in inflammatory arthritis [110]. Additional evidence indicates that SFs interact with T cells to suppress their activation and influence the course of RA [111].

### 5.2.2 Role of SFs in the humoral immune system

Humoral immunity involves a T-cell-mediated immune response where cytokines play a crucial role in the differentiation, development, activation, and proliferation of B cells by binding to specific receptors on the B-cell surface, including IL-1R, IL-2R, IL-4R, and IL-6R [112]. B-cell surface receptors also encompass B-cell receptor (BCR) complexes (mIg, Igα, and Igβ), complement receptors CR1 (CD35) and CR2 (CD21), Fc receptors (IgG), lipopolysaccharide, and human pokeweed mitogen [113]. B-cell surface antigens such as CD19, CD20, CD40, CD80, MHC antigens, ICAM-1, and antilymphocyte function-associated antigen-1 [114-116] bind to corresponding receptors, influencing signaling pathways, promoting B-cell differentiation, development, activation, proliferation, and facilitating crosstalk, which stimulates T-cell activation. Novel RA-SF cells stained positively for MH7A, IL-1R, ICAM-1, CD16, CD40, CD80, and CD95 [117]. Wang et al. [118] demonstrated that IL-6R antagonists reduced Th17 cell production mediated by IL-34-stimulated SFs in RA patients, thereby suppressing RA, indirectly suggesting high IL-6R expression in SFs correlates with disease activity. IgDR levels are significantly elevated in RA-SFs, contributing to the production of multiple inflammatory factors [119]. Characterization of SFs from synovial tissue isolated from patients with RA, chondromalacia, and healthy subjects revealed a notably higher proportion of CD90^+^ SFs [120]. Analysis of SF heterogeneity identified THY1^+^CD34^-^/THY1^+^CD34^-^HLA-DR^hi^ fibroblasts expressing genes associated with MHC class II molecules [10,11]. Significantly elevated expression of signaling lymphocytic activation molecule family member 8 (SLAM8) in TNF-α-stimulated RA-SFs promotes SF-mediated inflammation in RA [121]. Dong et al. [122] first reported the SLAM family as novel "do not eat me" receptors that inhibit macrophages from phagocytosing healthy blood cells.

B cells play a role in RA pathology by producing autoantibodies [123]. Human RA-SFs induce production of apoptosis-inducing ligand and proliferation-inducing ligand, promoting antibody maturation, class switching, and survival of plasma cells [124-126]. IL-6 produced by RA-SFs also enhances B-cell maturation, expansion, and survival [13-19]. VCAM1 on SFs regulates B-cell survival in synovial tissue in RA [127]. Additionally, interaction between B cells and RA-SFs in early RA increases IL-15R expression, supporting B-cell survival [128]. RA-SFs guide CXCR3^+^ plasma cells to the subsynovial layer, hastening RA progression [129]. Thrombin-cleaved bone bridge proteins bind to fibronectin, linking SFs to B cells and stimulating the production of inflammatory cytokines, worsening synovial inflammation [130]. TNF-α-activated RA-SFs promote B-cell maturation and maintenance through the induction of human VCAM1 (hVCAM1) and BAFF, accelerating joint damage [131]. Interactions between SFs and classical immune cells significantly amplify RA inflammation and bone destruction [132]. Previous studies have shown that interactions between SFs and activated B cells convert non-arthritic SFs into SFs with a proinflammatory RA-like phenotype [133]. IL-36R mediates the sustained activity of plasma cells and SF-mediated expansion of inflammatory Th1 and Th17 cells, diminishing protective immune responses [134]. Hypoxia-inducible factor 1α perpetuates interactions between RA-SFs and B cells [135].

## 6. Current status and outlook on targeted SF-based RA therapy

**Figure 2.**
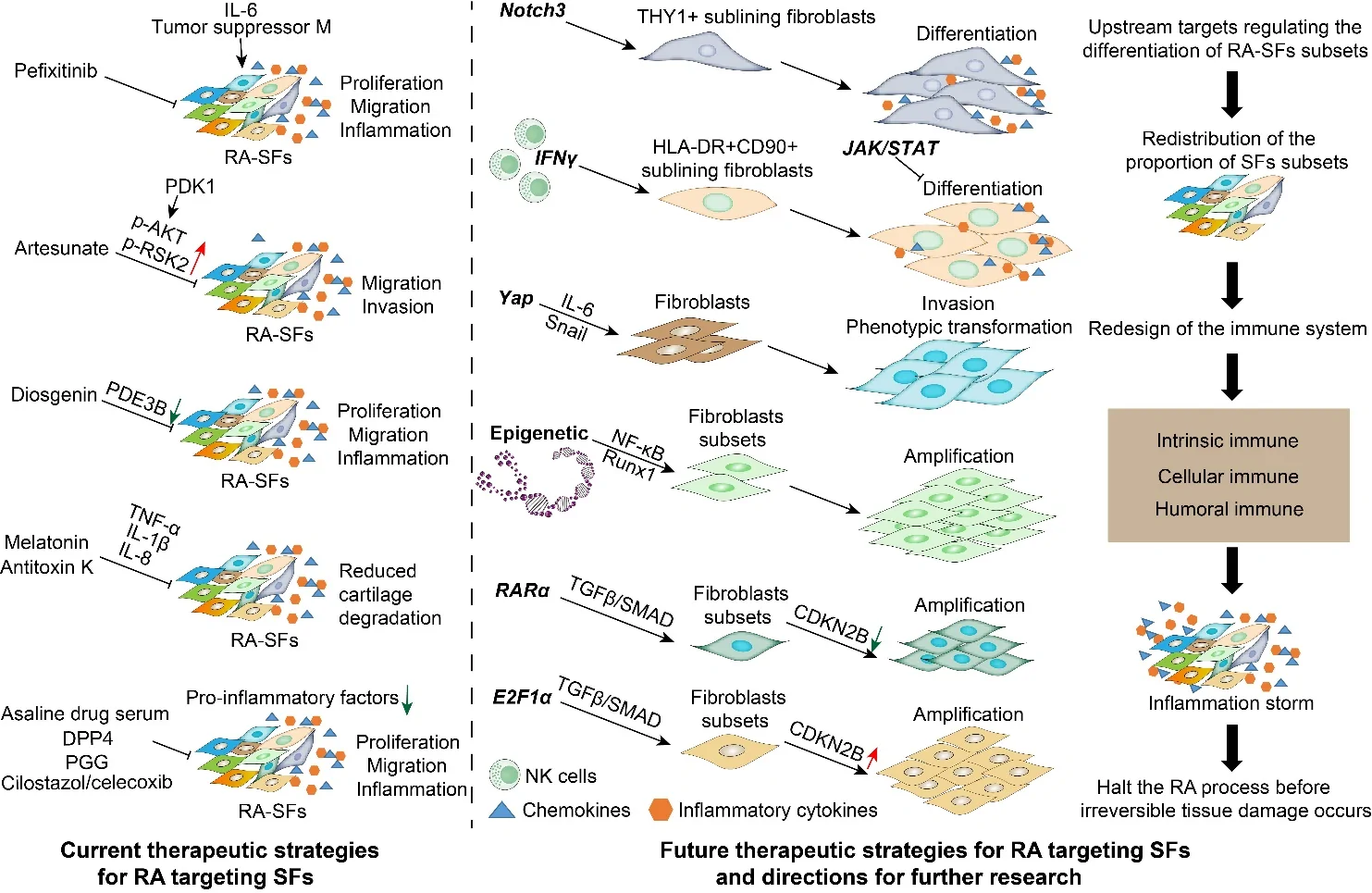
**Current and future therapeutic strategies for RA-targeting SFs and directions for further research**. Current clinical strategies for RA predominantly focus on broad anti-inflammatory treatments in advanced stages. These approaches aim to halt RA progression and alleviate symptoms by reducing inflammation, cytokine production, and cell proliferation. Future clinical directions and research in RA will primarily involve identifying factors that trigger heterogeneous transformation of SFs. Targeting and inhibiting these factors could potentially prevent inflammatory responses in synovial tissue, thereby halting classical immune system remodeling, preventing irreversible tissue damage, and reversing disease progression. RA (rheumatoid arthritis), SFs (synovial fibroblasts), DPP4 (dipeptidyl peptidase-4), PGG (pent-o-galloyl-β-d-glucose).

With a deeper grasp of RA's pathological mechanisms, therapies targeting SFs have become crucial in treating the condition. Yet, current SF-based treatments for RA provide limited benefits. This chapter will start by examining the current state of SF-focused therapies in RA, exploring the molecular mechanisms driving SF-mediated RA onset and progression, exploring potentially more effective treatment strategies, and looking ahead to future developments and prospects (Fig. 2).

### 6.1 Current status of targeted SF-based RA therapy

In recent years, treatments focusing on SFs have garnered increased attention due to their recognized pathogenic role in RA. Throughout the development of RA, RA-SFs exacerbate joint erosion and inflammation by adopting a destructive phenotype and increasing production of matrix-degrading enzymes and inflammatory cytokines. Consequently, targeting these specific properties of RA-SFs holds significant promise for developing targeted therapeutic strategies. However, current clinical approaches for RA primarily involve broad anti-inflammatory therapies in advanced stages, with no approved therapies directly targeting SFs (Fig. 2).

Preclinical studies have demonstrated that the Janus kinase (JAK) inhibitor pefixitinib effectively suppresses the inflammatory response induced by the tumor suppressor M and IL-6 trans-signaling in RA-SFs, as well as their proliferation and migration *in vitro* [136]. Artesunate inhibits the migration and invasion of RA-SFs by targeting pyruvate dehydrogenase kinase 1, which in turn suppresses activation of v-akt murine thymoma viral oncogene homolog and phosphorylation of ribosomal S6 kinase 2 (RSK2). This suggests that artesunate holds promise as a potential disease-modifying antirheumatic drug for RA [137]. Diosgenin suppresses the proliferation, migration, and inflammatory response of RA synoviocytes by downregulating phosphodiesterase 3B (PDE3B) [138]. Melatonin and antitoxin K mitigate the expression levels of TNF-α, IL-1β, and IL-8 in SFs, thereby reducing cartilage degradation and improving RA symptoms [83,139]. Asarin alleviates RA by inhibiting IL-17A, TNF-α, IFNγ, IL-6, TLR2, and TLR4 expression in RA-SFs [140]. Dipeptidyl peptidase-4 (DPP4, or CD26) attenuates RA symptoms by decreasing the production of proinflammatory cytokines in human RA-SFs [141]. Pent-o-galloyl-β-d-glucose alleviates inflammation in human RA-SFs and rat models of adjuvant-induced arthritis [142]. Cilostazol synergistically inhibits proinflammatory cytokines by activating IL-10 and suppressing cytokine signaling 3 in RA-SFs when used in combination with celecoxib for RA treatment [143].

These potential medications are designed to halt RA progression and relieve symptoms by targeting SFs to suppress their proliferation and production of inflammatory cytokines. Despite this, no significant positive clinical responses have been observed, underscoring the necessity for further research to discover more effective targeted therapeutic approaches. This requires gaining fresh insights into the molecular mechanisms underlying the development of RA.

### 6.2 Outlook on targeted SF-based RA therapy

Recent advances in single-cell sequencing, spatial transcriptomics, and epigenomic technologies are poised to revolutionize RA treatment [39]. These technologies enable differentiation and development studies of SFs, subset identification, and functional analysis. Identifying SF subsets with distinct functions, understanding phenotypic variations among SFs in individual joints, and exploring immune properties of RA-SFs have highlighted phases of relative disease stability in RA. Concurrently, investigating factors driving SF heterogeneous transformation may reshape conventional immune system paradigms, aiming to halt irreversible tissue damage and potentially reverse disease progression (Fig. 2).

Current research is progressively moving towards targeting the phenotypic reprogramming of SFs and their upstream targets as a novel therapeutic strategy to reverse immune dysregulation and reshape immune tolerance. Wei et al. [8] demonstrated that Notch3 signaling contributes to the differentiation of mural cells and THY1^+^ inferior fibroblasts, which is crucial in the development of inflammatory arthritis. Zhao et al. [144] identified that the HLA-DR^+^THY1^+^ phenotype indicates an activated state of SFs during RA inflammation induced by IFNγ and potentially by infiltrating leukocytes (e.g., activated NK cells). JAK inhibition targets inflammatory SFs that induce IL-6 production and may present antigens [144]. Previous studies have shown that targin alleviates RA inflammation by inhibiting the JAK/STAT pathway in RA-SFs [145]. Knocking down YAP prevents IL-6- or Snail-induced invasion of SFs, highlighting YAP's role in the phenotypic transformation of RA-SFs [146]. However, further investigation into the specific phenotypic alterations of these subsets is warranted. Armaka et al. [12] reported that epigenetically triggered genetic programs drive the expansion of a subpopulation of pathogenic RA SFs regulated by NF-κB and Runx1 transcription factors. Ainsworth et al. [147] identified critical transcription factors in individual RA-SFs through integrating transcriptomic and epigenomic data, characterizing clusters of RA cell lines with distinct transcriptional biology, which enhances understanding of disease heterogeneity [148]. Despite these advances, current therapeutic efficacy in treating RA remains limited, emphasizing the critical need for further development of targeted therapies.

Further research is crucial to identify upstream targets that regulate RA-SF subset differentiation, potentially leading to beneficial changes in the classical immune system and, ultimately, reversing RA progression before permanent damage occurs. RA, as an inflammatory autoimmune disease, often involves relapses and progressive deterioration at specific sites. Friscic et al. [15] highlighted that the complement system drives SFs to play a predominant role in inflammatory flares, which could also be linked to broader systemic immune responses. Investigating whether upstream targets influencing RA-SF subset differentiation can modulate the complement system by altering the ratios of SF subsets may help either promote or inhibit localized inflammatory responses. Additional studies are necessary to fully understand the shared immune characteristics between human RA and mouse models, including their underlying gene networks.

There remains a significant gap between current research and clinical needs in RA, necessitating deeper exploration in the future. Firstly, the molecular basis underlying phenotypic reprogramming in SF subpopulations is still poorly understood. Secondly, our knowledge of the upstream targets that govern the differentiation of RA-SF subpopulations is limited, hindering a comprehensive understanding of RA pathogenesis and the development of novel therapies. Thirdly, while research suggests that altering the ratios of SF subpopulations may influence the complement system, the specific molecular mechanisms involved remain unclear, thus limiting our understanding of RA's inflammatory processes. Fifthly, despite insights gained from laboratory research into RA pathogenesis, translating these findings into effective clinical therapies remains challenging. Therefore, future studies should focus on elucidating the molecular mechanisms of SF phenotypic reprogramming and exploring new targeted strategies to intervene in these mechanisms to prevent or reverse RA progression. These therapies should target SF subpopulation phenotypic reprogramming, upstream regulatory factors, or complement system regulation to effectively treat RA. Additionally, future research should prioritize translational medical research to accelerate the development and application of new therapies from the laboratory to clinical settings.

## 7. Conclusions

This study has uncovered that reallocating heterogeneous SF subpopulation ratios increases the presence of immune-effective SF subsets, perpetuating the inflammatory state in RA patients. These subsets exhibit robust immune characteristics, functioning akin to immune cells and playing a pivotal role in RA pathogenesis. Therefore, targeting phenotypic reprogramming of SF subpopulations, upstream regulators, and immune system remodeling could offer novel avenues for RA treatment. Future research should investigate whether upstream targets can modulate the complement system by adjusting SF subpopulation ratios, thereby influencing local inflammation. This study lays the groundwork for innovative targeted therapies in RA aimed at reversing immune dysregulation, reshaping the classical immune system, and providing timely interventions to prevent irreversible RA damage. In conclusion, our analysis provides a fresh perspective on the role of SFs in RA and holds promise for transforming RA treatment strategies.
